# Associations between external beam radiotherapy and overall survival in patients with gallbladder cancer: A population-based study

**DOI:** 10.3389/fpubh.2022.1012142

**Published:** 2022-10-10

**Authors:** Jiazhao Song, Xiaoli Kang, Yupeng Di, Gang Ren, Yingjie Wang

**Affiliations:** ^1^Graduate School of Hebei North University, Zhangjiakou, China; ^2^Department of Radiotherapy, Air Force Medical Center PLA, Beijing, China

**Keywords:** radiotherapy, survival benefits, gallbladder cancer, SEER database, treatment

## Abstract

**Background:**

There is a lack of studies regarding radiotherapy (RT) in patients with gallbladder cancer (GBC) on the survival benefit after surgery and nonsurgical treatment. Therefore, this study evaluated the impact of external beam RT on the overall survival (OS) of patients with GBC in a real-world setting.

**Methods:**

Patients with GBC enrolled from the Surveillance, Epidemiology, and End Results (SEER) database were examined through Kaplan–Meier survival curves and multivariable Cox regression analyses.

**Results:**

A total of 7,866 patients with GBC were screened for the current analysis, of whom 2,130 (27.1%) did not undergo RT or surgery, 209 (2.7%) underwent RT, 4,511 (57.3%) underwent surgery, and 1,016 (12.9%) underwent both RT and surgery. The median OS times were 4 months, 8 months, 16 months, and 22 months (*p* < 0.0001). OS was significantly different between adjuvant RT (*p* = 0.0002) and palliative RT (*p* < 0.0001). Multifactorial analysis (controlling for age, sex, year of diagnosis, marital status, race, grade, and stage) showed that both adjuvant RT (surgery and adjuvant RT vs. surgery alone; HR, 0.75; 95% CI, 0.69–0.82, *p* < 0.001) and palliative RT (RT alone vs. no treatment; HR, 0.80; 95% CI, 0.69–0.92, *p* = 0.003) had a significant impact on patient OS. The results remained stable following sensitivity analyses.

**Conclusion:**

The study results indicate that adjuvant and palliative radiation treatment was associated with a survival benefit. GBC patients can derive a survival benefit from external beam RT.

## Introduction

Gallbladder cancer (GBC) is the most common type of biliary tract malignancy and is relatively rare worldwide but is highly malignant and lethal (1). In 2021, an estimated 4,310 patients died from GBC and other bile duct cancers in the United States, and ~40% of new cases will be GBC. The annual mortality rate is approximately 1,700 per year (2). Surgical resection or segment resection of the liver is considered the only curative modality in GBC (3). However, GBC is very insidious in onset and difficult to diagnose; surgical resection is recommended in the early stage for patients with GBC (4, 5). Nevertheless, even in patients who can undergo surgical resection, positive margins are common, and the curative resection rates range from only 10 to 30% (6); patients often have poor surgical results, with most patients experiencing postoperative recurrence or metastasis, with a 5-year survival rate between 18 and 34% (7, 8).

Postoperative adjuvant therapy plays an important role and is used to curb disease progression in inoperable patients. For patients who have lost the opportunity to undergo resection, the overall prognosis is poor. Therapeutic options are limited to palliative treatment to relieve symptoms and improve survival. Therefore, for patients with GBC, the combination of non-surgical (radiotherapy (RT) or chemotherapy) and surgical treatments becomes more critical in both the limited and progressive stages of the disease. However, chemotherapy has shown limited efficacy in patients with GBC. In patients with locally advanced or metastatic biliary tract cancer (BTC), including GBC, a phase 3 trial reported a median overall survival (OS) of 8.1 months in the BTC group treated with single-agent chemotherapy, and even with a two-drug combination chemotherapy, the OS was only 11.7 months in the BTC group (9). Although RT is recommended as a local treatment modality according to the National Comprehensive Cancer Network (NCCN) guidelines, prior population-based studies have shown a general lack of effective utilization of RT for hepatobiliary malignancies (10).

In addition, there was controversy regarding the value of radiotherapy in GBC, and studies at Memorial Sloan-Kettering Cancer Center point to a higher incidence of synchronous distant metastases in GBC than regional spread, making it difficult for surgical patients to benefit from local treatment (11). However, most previous studies have focused on retrospective clinical studies with small samples, which seem to indicate a potential benefit from chemotherapy and/or RT, while there are few studies with large samples (12–17). Most of the existing studies have focused on postoperative adjuvant external beam RT (16, 18, 19), and studies on palliative external beam RT are still lacking. Because of the rarity of GBC, it may prove to be difficult to accrue sufficient numbers of patients for a large-scale clinical trial, and the actual benefit of RT has not been well established. As a result, clinicians currently have limited evidence to rely on when attempting to comprehensively assess whether external beam RT is beneficial to GBC patients. Nevertheless, the impact of RT on survival in patients with cancer is undeniable and thus necessitates additional research.

The purpose of this study was to use the Surveillance, Epidemiology, and End Results (SEER) database to examine the role of external beam radiation in postoperative and inoperable patients with GBC at a population-based level.

## Methods

### Data sources

This study was conducted on cancer patients enrolled from a cancer database representing approximately 48% of the U.S. population that the research plus data from 18 registries (2000-2018) (https://seer.cancer.gov/data/). The GBC data were obtained from SEER^*^Stat software version 8.3.9.2. The SEER initiative has standardized data collection practices and provides only de-identified information, so this study did not require research ethical or institutional review board approval. The researchers obtained approval to access the database (username: 15548-Nov2020).

### Population study cohort

GBC was identified with the SEER primary site code of C23.9 (International Classification of Diseases for Oncology ICD-0-3) between 2004 and 2015. The time of diagnosis was chosen from 2004 to 2015 because the reference tumor stage we selected was based on the “SEER Combined Summary Stage” summarized from 2004-2017. This study endpoint of 2015 was chosen to allow for a minimum of 3 years of follow-up because the last data entry point for this cohort was December 2018.

External beam RT was chosen (“Radiation recode” code was “Beam radiation”) as the modality to receive radiation therapy. Patients received surgical treatment, which was defined as at least cholecystectomy or any other procedure with more extensive resection. This study included patients with “RX Summ Surg Prim Site (1998+)” field codes “30, 40, 50, 60, and 90” when diagnosed in 2004 and 2015 (20). The exclusion criteria for GBC patients were the not first diagnosed malignancy (First malignant primary indicator: No), without positive histology (Diagnostic Confirmation: not coded as positive histology) and diagnosed by autopsy or death (Type of Reporting Source: autopsy only, death certificate only). This study also excluded patients who were younger than 20 years of age, whose race and marital status were unknown, whose cancer stage was unknown, and who survived for <1 month following the diagnosis. Patients with an unknown surgery in primary site statuses (RX Summ Surg Prim Site code “99”) were also excluded. Regarding RT, we excluded patients who were recommended to undergo RT but had an unknown status of whether this treatment was administered.

Finally, based on surgery and RT information in the SEER database, patients were grouped after redefining the type of treatment: the postoperative adjuvant RT group included surgery only and RT only; the palliative RT group included non-RT and non-surgery.

### Statistical analysis

Descriptive analysis was applied to the data of all participants. Categorical variables are expressed as proportions and percentages (%). Categorical variables were compared by the chi-square test or Fisher's exact test. Univariate and multivariable Cox regression analyses were performed to assess the associations between treatment type and covariates with overall mortality. Variables with *p* < 0.05 in univariate Cox regression analysis as well as important clinical variables and confounders were entered into the multivariate model. Survival curves were plotted *via* the Kaplan–Meier method, and the log-rank test was used to compare each treatment group.

Subgroup analyses examined the relationship between treatment status and survival outcomes according to potentially confounding covariates. Interaction tests within Cox proportional hazards models were implemented to compare hazard ratios (HRs) between the analyzed subgroups.

Propensity score matching (PSM) was used to adjust for confounding factors and improve comparability between groups in this retrospective study (21). Age, sex, year of diagnosis, marital status, race, stage, grade, tumor size, and chemotherapy status were adjusted by the logistic regression model to calculate the propensity score. The patients were then matched in a 1:1 ratio. The matching algorithm was applied with a caliper width of 0.2 (22). The degree of PSM was estimated using standardized mean differences (SMDs). Optimal balance on a parameter is generally achieved when the SMD is equal to or below 0.1. The standardized mortality ratio weighting (SMRW) and overlap weighting (OW) model that unified the distribution of the risk factors for both groups was used to confirm the robustness of the results (23, 24).

Statistical analyses were performed with R v.4.4.1 statistical software (http://www.R-project.org, The R Foundation, Vienna, Austria), Statistical Package for Social Sciences (SPSS) software Version 25.0 (IBM, Armonk, NY, USA), and Free Statistics software (v.1.3, Beijing, China). Two-tailed *p-*values of < 0.05 were considered statistically significant. HRs are presented with 95% confidence intervals (CIs).

## Results

### Basic features of the patients

**Figure 1:**
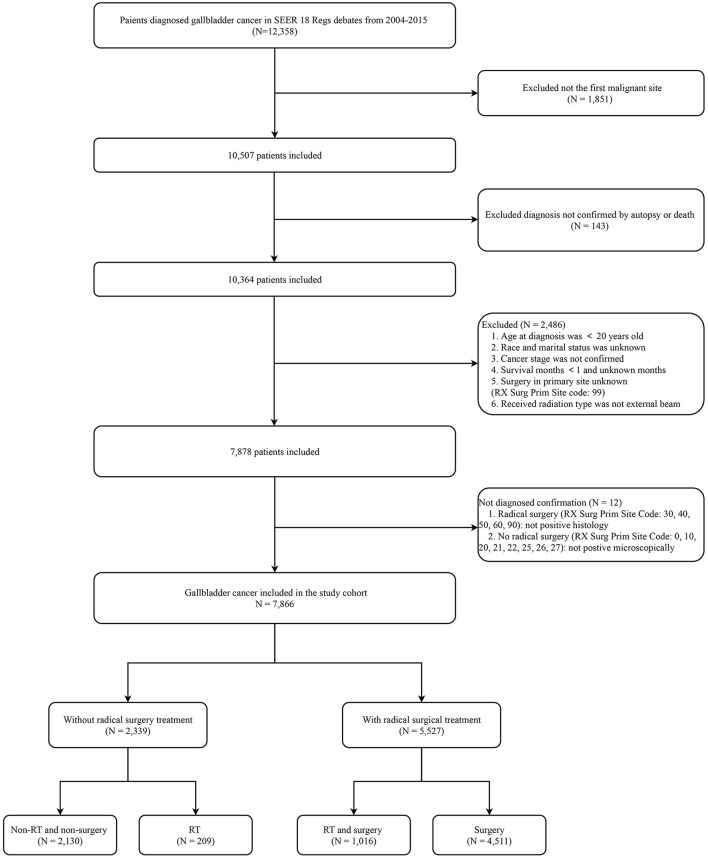
Flow chart for study enrollment. RT, radiotherapy; SEER, Surveillance, Epidemiology, and End Results.

Based on the criteria listed above, 12,358 patients with GBC were identified from the SEER database between 2004 and 2015. A total of 7,866 patients remained and were included in the final analysis. A flow chart depicting the study's detailed inclusion and exclusion criteria is shown in Figure 1.

The baseline demographic characteristics of all included patients are summarized by treatment category in Table 1. Overall, a total of 4,511 (57.3%) patients underwent only surgery, 209 (2.7%) patients underwent only RT, 2,130 (27.1%) patients underwent neither RT nor surgery, and 1,016 (12.9%) patients underwent both RT and surgery. There were significant differences in six variables (age, sex, race, year of diagnosis, marital status, and stage) that were associated with the type of treatment (*p* < 0.001). A total of 51.9% of the target population was below 70 years old; 70.5% of the patients were female, and 75.7% were white. Slightly more patients were married in the four different groups (*p* < 0.001). Few patients (46.1%) were diagnosed from 2004 through 2009, but most (53.9%) were diagnosed within the next six years (2010–2015). There were more patients with a distant stage (43.4%) at the time of diagnosis than with regional (21.9%) or localized (34.7%) disease (*p* < 0.001). The other three variables (grade, tumor size, and chemotherapy) were also significantly different among the four treatment groups (*p* < 0.001), although some data were unknown.

**Table 1:** Baseline characteristics of gallbladder cancer patients.

**Variables**	**Patient and tumor characteristics**					
	**All** ***n =* 7,866** **(100%)**	**Non-RT and non-surgery** ***n =* 2,130 (27.1%)**	**RT** ***n =* 209 (2.7%)**	**Surgery** ***n =* 4,511** **(57.3%)**	**RT and surgery** ***n =* 1,016** **(12.9%)**	*p*-value*
**Age**						<0.001
<70	4,080 (51.9)	1,156 (54.3)	140 (67)	2,143 (47.5)	641 (63.1)	
≥70	3,786 (48.1)	974 (45.7)	69 (33)	2,368 (52.5)	375 (36.9)	
**Sex**						0.001
Female	5,547 (70.5)	1,445 (67.8)	140 (67)	3,260 (72.3)	702 (69.1)	
Male	2,319 (29.5)	685 (32.2)	69 (33)	1,251 (27.7)	314 (30.9)	
**Year of diagnosis**						<0.001
2004–2009	3,623 (46.1)	897 (42.1)	81 (38.8)	2,156 (47.8)	489 (48.1)	
2010–2015	4,243 (53.9)	1,233 (57.9)	128 (61.2)	2,355 (52.2)	527 (51.9)	
**Marital status**						<0.001
Married	4,186 (53.2)	1,137 (53.4)	123 (58.9)	2,301 (51)	625 (61.5)	
Others	3,680 (46.8)	993 (46.6)	86 (41.1)	2,210 (49)	391 (38.5)	
**Race**						<0.001
White	5,956 (75.7)	1,540 (72.3)	139 (66.5)	3,522 (78.1)	755 (74.3)	
Others	1,910 (24.3)	590 (27.7)	70 (33.5)	989 (21.9)	261 (25.7)	
**Stage**						<0.001
Distant	3,416 (43.4)	1,720 (80.8)	138 (66)	1,277 (28.3)	281 (27.7)	
Localized	2,726 (34.7)	75 (3.5)	7 (3.3)	2,310 (51.2)	334 (32.9)	
Regional	1,724 (21.9)	335 (15.7)	64 (30.6)	924 (20.5)	401 (39.5)	
**Grade**						<0.001
I	874 (11.1)	56 (2.6)	5 (2.4)	709 (15.7)	104 (10.2)	
II	2,453 (31.2)	231 (10.8)	29 (13.9)	1,753 (38.9)	440 (43.3)	
III	2,322 (29.5)	366 (17.2)	49 (23.4)	1,530 (33.9)	377 (37.1)	
IV	168 (2.1)	31 (1.5)	1 (0.5)	104 (2.3)	32 (3.1)	
Unknown	2,049 (26.0)	1,446 (67.9)	125 (59.8)	415 (9.2)	63 (6.2)	
**Tumor size**						<0.001
<5 cm	3,119 (39.7)	385 (18.1)	54 (25.8)	2,195 (48.7)	485 (47.7)	
≥5 cm	1,275 (16.2)	402 (18.9)	55 (26.3)	628 (13.9)	190 (18.7)	
Unknown	3,472 (44.1)	1,343 (63.1)	100 (47.8)	1,688 (37.4)	341 (33.6)	
**Chemotherapy**						<0.001
No/Unknown	4,627 (58.8)	996 (46.8)	45 (21.5)	3,420 (75.8)	166 (16.3)	
Yes	3,239 (41.2)	1,134 (53.2)	164 (78.5)	1,091 (24.2)	850 (83.7)	

RT, radiotherapy.

Categorical variables are expressed as frequencies outside the parentheses and percentages in parentheses.

*p-values based on Pearson's chi-square test or Fisher's exact test.

### Univariate survival analysis

The greatest reduction in the risk of mortality was seen in patients treated with surgery and RT (HR = 0.27; 95% CI, 0.25–0.29, *p* < 0.001), followed by surgery (HR, 0.31; 95% CI, 0.30–0.33, *p* < 0.001), RT (HR, 0.68; 95% CI, 0.58–0.79, *p* < 0.001), and no RT or surgery, as shown in Table 2. Increasing age, marital status classified as other (divorced, separated, single, unmarried or domestic partner, and widowed), regional or distant stage, grade between I and IV, tumor size ≥ 5 cm, and chemotherapy were associated with a significantly greater risk of mortality (HR > 1, *p* < 0.001). No significant increase in mortality was seen based on sex (male HR, 1.04; 95% CI, 0.99–1.10), year of diagnosis (2010–2015 HR, 0.96; 95% CI, 0.91–1.00) or other races [1.01 (95% CI, 0.95–1.07)].

**Table 2:** Univariate and multivariate Cox proportional hazards regression analyses for overall survival.

**Variable**	**Univariate**		Multivariate adjusted*	
	**HR (95% CI)**	***p-*value**	**HR (95% CI)**	***p-*value**
**Treatment type**				
Non-RT and non-surgery	Ref		Ref	
RT	0.68 (0.58–0.79)	<0.001	0.77 (0.66–0.89)	<0.001
Surgery	0.31 (0.30–0.33)	<0.001	0.52 (0.48–0.56)	<0.001
RT and surgery	0.27 (0.25–0.29)	<0.001	0.42 (0.38–0.46)	<0.001
**Age**				
<70	Ref		Ref	
≥70	1.33 (1.27–1.40)	<0.001	1.42 (1.35–1.49)	<0.001
**Sex**				
Female	Ref		Ref	
Male	1.04 (0.99–1.10)	0.126	1.10 (1.04–1.16)	<0.001
**Year of diagnosis**				
2004–2009	Ref		Ref	
2010–2015	0.96 (0.91–1.00)	0.069	0.95 (0.90–1.00)	0.047
**Marital status**				
Married	Ref		Ref	
Others	1.16 (1.10–1.21)	<0.001	1.18 (1.12–1.24)	<0.001
**Race**				
White	Ref		Ref	
Others	1.01 (0.95–1.07)	0.782	1.02 (0.96–1.08)	0.517
**Stage**				
Localized	Ref		Ref	
Regional	2.34 (2.18–2.50)	<0.001	2.41 (2.24–2.59)	<0.001
Distant	4.13 (3.88–4.39)	<0.001	3.66 (3.40–3.94)	<0.001
**Grade**				
I	Ref		Ref	
II	1.52 (1.39–1.67)	<0.001	1.36 (1.24–1.49)	<0.001
III	2.43 (2.22–2.67)	<0.001	1.85 (1.68–2.03)	<0.001
IV	2.51 (2.09–3.00)	<0.001	1.94 (1.61–2.33)	<0.001
Unknown	3.13 (2.85–3.44)	<0.001	1.43 (1.29–1.58)	<0.001
**Tumor size**				
<5 cm	Ref		Ref	
≥5 cm	1.76 (1.64–1.89)	<0.001	1.29 (1.20–1.39)	<0.001
Unknown	1.82 (1.72–1.92)	<0.001	1.36 (1.29–1.44)	<0.001
**Chemotherapy**				
No/Unknown	Ref		Ref	
Yes	1.17 (1.11–1.22)	<0.001	0.69 (0.65–0.74)	<0.001

HR, hazard ratio; CI, confidence interval; RT, radiotherapy; Ref, reference.

The hazard ratio (HR) and 95% confidence interval (CI) were calculated for survival status with respect to treatment status using Cox proportional hazards models. Both non-adjusted and multivariate-adjusted models were implemented.

*Adjusted for age, sex, year of diagnosis, marital status, race, stage, grade, tumor size, and chemotherapy status.

**Figure 2:**
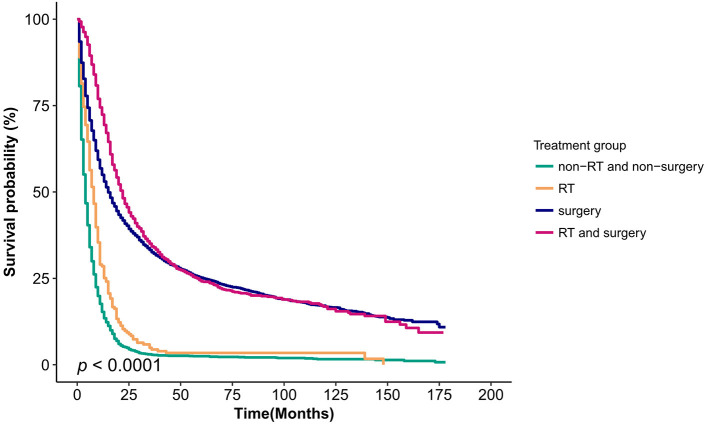
Kaplan–Meier survival curves for the four treatment category groups (non-RT and non-surgery, RT and surgery, RT, and surgery) for patients with gallbladder cancer. RT, radiotherapy.

The greatest survival benefit was seen for patients treated with RT and surgery, with a median OS time of 22.00 months (95% CI, 20.25-23.75) compared with 16.00 months (95% CI, 15.02–16.98; *p* < 0.001) for patients treated with surgery alone (Table 3). The median OS time was 8.00 months (95% CI, 6.82-9.18) for patients treated with RT alone compared with 4 months (95% CI, 3.74–4.26) for patients treated with non-RT or non-surgical procedures (*p* < 0.001) (Table 3). Figure 2 shows the Kaplan–Meier curves of OS for different treatments. The median OS times were 4 months, 8 months, 16 months, and 22 months (*p* < 0.0001) (Table 4).

**Table 3:** Cox regression analysis for overall survival of the two treatment category groups.

**Treatment categories**	**Univariate analysis**	***p-*value**	Multivariate analysis*	***p-*value**
	**HR (95% CI)**		**HR (95% CI)**	
**Palliative treatment group**				
Non-RT and non-surgery	Ref		Ref	
RT	0.68 (0.59–0.79)	<0.001	0.80 (0.69–0.92)	0.003
**Adjuvant treatment group**				
Surgery	Ref		Ref	
RT and surgery	0.86 (0.80–0.93)	<0.001	0.75 (0.69–0.82)	<0.001

HR, hazard ratio; CI, confidence interval; RT, radiotherapy; Ref, reference.

HRs and 95% CIs were calculated for survival status with respect to radiation status using Cox proportional hazards models. Both nonadjusted and multivariate-adjusted models were implemented.

*Adjusted for age, sex, year of diagnosis, marital status, race, stage, grade, tumor size, and chemotherapy status.

**Table 4:** Median overall survival time^*^.

**Treatment group**	**Median survival (95% CI)**
Non-RT and non-surgery	4.00 (3.74–4.26)
RT	8.00 (6.82–9.18)
Surgery	16.00 (15.02–16.98)
RT and surgery	22.00 (20.25–23.75)

CI, confidence interval; RT, radiotherapy.

*Log–rank test, p <0.0001.

### Multivariate survival analysis

After adjustment in Cox proportional hazards regression analysis, surgery and RT also had the most significant OS benefit (HR, 0.42; 95% CI 0.38–0.46, *p* < 0.001), followed by surgery (HR = 0.52; 95% CI 0.48–0.56, *p* < 0.001), RT (HR = 0.77; 95% CI 0.66–0.89, *p* < 0.001) and no treatment (reference group). Regional (HR, 2.41; 95% CI, 2.24–2.59, *p* < 0.001) and distant (HR, 3.66; 95% CI, 3.40–3.94, *p* < 0.001) stages were associated with worse outcomes than localized disease (Table 2). Age above 70, male sex, other marital status (divorced, separated, single, unmarried or domestic partner, and widowed), regional or distant stage, grade I to IV, and tumor size ≥ 5 cm were associated with a significantly high risk of poor prognosis (HR > 1, *p* < 0.001). Only other races (Black, American Indian/AK native, Asian/Pacific Islander) were not associated with prognosis (HR, 1.02; 95% CI, 0.96–1.08, *p* = 0.517). Patients with cancer who received chemotherapy and were diagnosed between 2010 and 2015 (HR <1, *p* < 0.05) had improved OS, as shown in Table 2.

### Sensitivity analysis

Although we observed statistically significant interactions for sex and tumor grade in subgroup analyses, the overall HR is a true representation of the effects of RT. The subgroup-specific HRs in the current study suggest that the effective values representing associations between treatment and OS maintained stability within subgroup analyses (Supplementary Figure 1).

To further corroborate the findings from univariate and multivariate Cox proportional hazards regression analyses, PSM analyses were performed as described in the statistical analysis. All the SMD values in the model of the SMRW and OW cohorts were < 0.1 and were far less than those in the unmatched cohort (Supplementary Figure 2). The overall direction in the SMRW (HR <1, *p* < 0.05) and OW (HR <1, *p* < 0.05) models reflected in the treatment groups at different stages was consistent and significant. A significant improvement in survival was found in the treatment group in the entire patient cohort after adjusting for age, sex, year of diagnosis, marital status, race, stage, grade, tumor size, status of chemotherapy (adjuvant treatment group; HR = 0.80; 95% CI, 0.73–0.87, *p* < 0.001) (palliative treatment group; HR = 0.81; 95% CI, 0.70–0.94, *p* = 0.007) and matched result (adjuvant treatment group; HR = 0.74; 95% CI, 0.66–0.81, *p* < 0.001) (palliative treatment group; HR = 0.78; 95% CI, 0.64–0.95, *p* = 0.012). In multivariate analysis, non-RT and non-surgical treatments were found to be associated with significantly improved survival compared to RT (HR = 0.78; 95% CI, 0.67–0.91, *p* = 0.001) and propensity score analysis (HR = 0.80; 95% CI, 0.69–0.93, *p* = 0.004) (Table 5).

**Table 5:** Association between treatment group and overall mortality.

**Model**	**Surgery vs. RT and surgery (Adjuvant treatment group**		**Non-RT and non-surgery vs. RT (Palliative treatment group)**	
	**HR (95% CI)**	***p-*value**	**HR (95% CI)**	***p-*value**
Propensity score adjusted*	0.80 (0.73–0.87)	<0.001	0.81 (0.70–0.94)	0.007
Propensity score matched	0.74 (0.66–0.81)	<0.001	0.78 (0.64–0.95)	0.012
SMRW	0.74 (0.69–0.80)	<0.001	0.79 (0.69–0.92)	0.002
OW	0.79 (0.69–0.89)	<0.001	0.79 (0.64–0.97)	0.028
Multivariate adjusted after PSM*	0.71 (0.64–0.78)	<0.001	0.75 (0.61–0.92)	0.005

RT, radiotherapy; HR, hazard ratio; CI, confidence interval; SMRW, standardized mortality ratio weighting; OW, overlap weighting; PSM, propensity score matching.

Cox proportional hazards model comparing the quartiles in different models to determine whether the overall HR is a true representation of the effects of adjuvant RT and palliative RT.

^*^Adjusted age, sex, year of diagnosis, marital status, race, stage, grade, tumor size, and chemotherapy status after propensity score matching.

After PSM, to verify the stability of the results and analyze the associations between RT and survival benefit, the multivariate Cox model adjusted all variables again. Patients treated with both RT and surgery had a significantly improved survival rate compared to patients treated with surgery only (HR = 0.71; 95% CI, 0.64–0.78, *p* < 0.001). RT was associated with better outcomes than non-RT and non-surgery (HR, 0.75; 95% CI, 0.61–0.92, *p* = 0.005) (Table 5).

## Discussion

The present population-based study included more than 7,800 patients diagnosed between 2004 and 2015, providing compelling evidence after adjustment for multivariate and sensitivity analyses. In both adjuvant and palliative care, RT was associated with improved median survival rates and was likewise associated with a reduction in mortality risk in GBC patients. Sensitivity analyses demonstrated that the OS benefit of radiation was maintained even when there was an imbalance of detrimental variable evidence and despite the emergence of interactions in the grade variables of the subgroup. The SEER data are based on the U.S. population, so they are likely more reflective of the population experience compared with previously published data focused primarily on patients treated at cancer centers. This study suggests that both adjuvant and palliative RT are associated with improved OS and can be used as a means to improve survival in patients in a real-world setting. Palliative RT has a more pronounced advantage in improving OS, whereas adjuvant radiotherapy appears to improve only early survival in patients with GBC. Ultimately, because of the high mortality of GBC, although RT prolonged survival, patients were not cured of their disease (Figure 2).

In the treatment of patients with GBC, the 5-year survival rate of surgical resection is <40% (7, 8). Therefore, studies with adjuvant chemoradiotherapy are needed to improve the cure rates for this devastating disease. Chemotherapy with gemcitabine and cisplatin is the landmark of non-surgical studies (9), and concurrent chemoradiotherapy is feasible, well tolerated and worth exploring in future studies (19). However, the current disappointing prognosis of GBC in terms of treatment deserves focused attention, the main issue being the effectiveness of radiotherapy, which still lacks large-scale studies in patients with different stages of GBC.

Since ~ 70–90% of patients with GBC develop unresectable disease, it is critical to determine the best treatment for these patients (4). Compared to most of the published studies on this topic, our results confirm and extend the previous study on the role of external beam RT in postsurgical and inoperable patients with GBC. Hyder et al. (18) studied the impact of adjuvant external beam RT on survival in patients with surgically resected gallbladder adenocarcinoma from the SEER database between 1988 and 2009. They found that the median survival was 15 months in patients with GBC who received external beam RT after surgery. However, chemotherapy was not included in the covariate analysis, even though the results after PSM were still unstable. Another study on SEER data suggested that adjuvant RT improves overall survival after surgery in patients with regional metastases, but that investigation only retrospectively analyzed GBC with regional metastases and did not include patients with distant metastases that could tolerate surgery (25). Postoperative local recurrence in surgical patients is a major cause of treatment failure and death in GBC (26). The efficacy of local treatment should be analyzed comprehensively in the population, and our study expands on the above study by refining the evidence of RT in patients with distant metastases while chemotherapy was included in the analysis as a covariate and comprehensively analyzing the survival benefit of RT-directed treatment modalities. The median survival of patients with unresectable disease is ~ 8–12 months after chemotherapy (9, 27–29). The results of this study showed that the median survival of patients treated with RT only was 8 months, which was similar to the survival benefit seen with chemotherapy. However, regarding the role of external beam radiation, there is insufficient evidence to support palliative treatment in patients with GBC, since it was only used in clinical trials in Europe and is not widespread in clinical treatment (30).

Early studies on small samples have demonstrated the benefits of palliative RT. Survival rates were improved compared to those of historical controls, and palliative RT may be a safe procedure to help relieve symptoms such as jaundice, pain, and itching in patients with gallbladder carcinoma (26, 31, 32). Most of the metastatic GBC included in our study was distributed between RT and untreated patients, which is well represented in the illustration of palliative evidence. Median OS survival and post-PSM multivariate Cox regression were compared for RT versus no surgery without RT, illustrating that palliative RT significantly improved survival and prognosis (Tables 4, 5). A recent study showed that combination therapy consisting of intraarterial chemotherapy plus external beam RT might further improve these results (33).

Our study exemplifies the survival benefit of radiotherapy for GBC at different stages. Studies on the underlying molecular mechanisms of this phenomenon have also had mixed results when focusing mainly on the sensitivity of RT. Although it is a common deletion in biliary tract cancer, human cell-based studies confirm that radiosensitivity does not correlate with the expression status of p53 (34). However, activation of the serine/threonine kinase enzyme AKT in human cholangiocarcinoma was associated with increased radioresistance, thus suggesting to us that decreasing AKT activation could reduce radioresistance and increase sensitivity to RT (35). Although it seems feasible to extrapolate this result to GBC, as the primary site of both gallbladder and bile duct cancers is the same biliary system, studies on the underlying radiation mechanisms of GBC are still lacking and need to be further explored in the future.

In addition to the substantial strengths of the current study, the findings are highly generalizable. However, our study was a retrospective study based on the publicly available SEER database. There may be potentially confounding variables associated with both outcomes and radiation treatment. Therefore, it was not possible to analyze in-depth the impact of surgery in combination with other treatment modalities on patient prognosis. Therefore, the data were used PSM in sensitivity analyses to control for differences between treatment groups whenever possible. As such, multivariate Cox regression analysis after PSM showed that the results of this study remained stable and significant (Table 5).

## Conclusion

The present study indicates that radiation is beneficial for OS. It is worth considering either adjuvant or palliative radiation in GBC patients. The results of the current investigation will inform prospective, highly informative, and influential clinical studies. In addition, to better increase the survival benefit of this devastating disease, the combination of surgery and chemoradiotherapy is a promising strategy for future research.

## Data availability statement

Publicly available datasets were analyzed in this study. This data can be found here: https://seer.cancer.gov/.

## Author contributions

YW and JS conceived and designed the study. YD, XK, and GR collected data and analyzed the literature. JS, XK, and YD analyzed and interpreted the data and drafted the manuscript. All authors contributed to the critical revision, editing, and approval of the final manuscript.

## Conflict of interest

The authors declare that the research was conducted in the absence of any commercial or financial relationships that could be construed as a potential conflict of interest.

## Publisher's note

All claims expressed in this article are solely those of the authors and do not necessarily represent those of their affiliated organizations, or those of the publisher, the editors and the reviewers. Any product that may be evaluated in this article, or claim that may be made by its manufacturer, is not guaranteed or endorsed by the publisher.
