# Supplementary material for: Associations between external beam radiotherapy and overall survival in patients with gallbladder cancer: A population-based study
Source: Front Public Health. 2022 Oct 10;10:1012142. doi: 10.3389/fpubh.2022.1012142 (PMC9614712; doi:10.3389/fpubh.2022.1012142)
Supplement: Supplementary file 1 [file Table_1.DOCX]

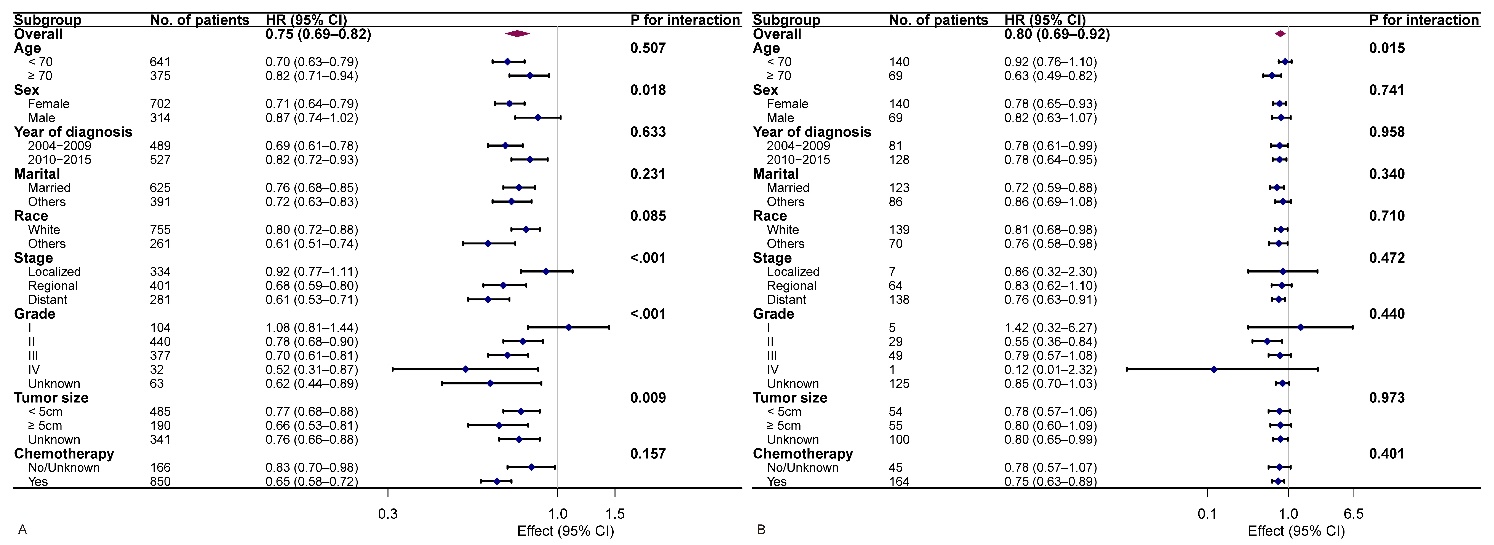


**Supplementary Figure 1.** Associations between radiotherapy and mortality according to previous characteristics A (RT and surgery vs. surgery), B (non-RT and non-surgery vs. RT). Each stratification was adjusted for potentially confounding factors (i.e., age, sex, year of diagnosis, marital status, race, stage, tumor size, and chemotherapy status).


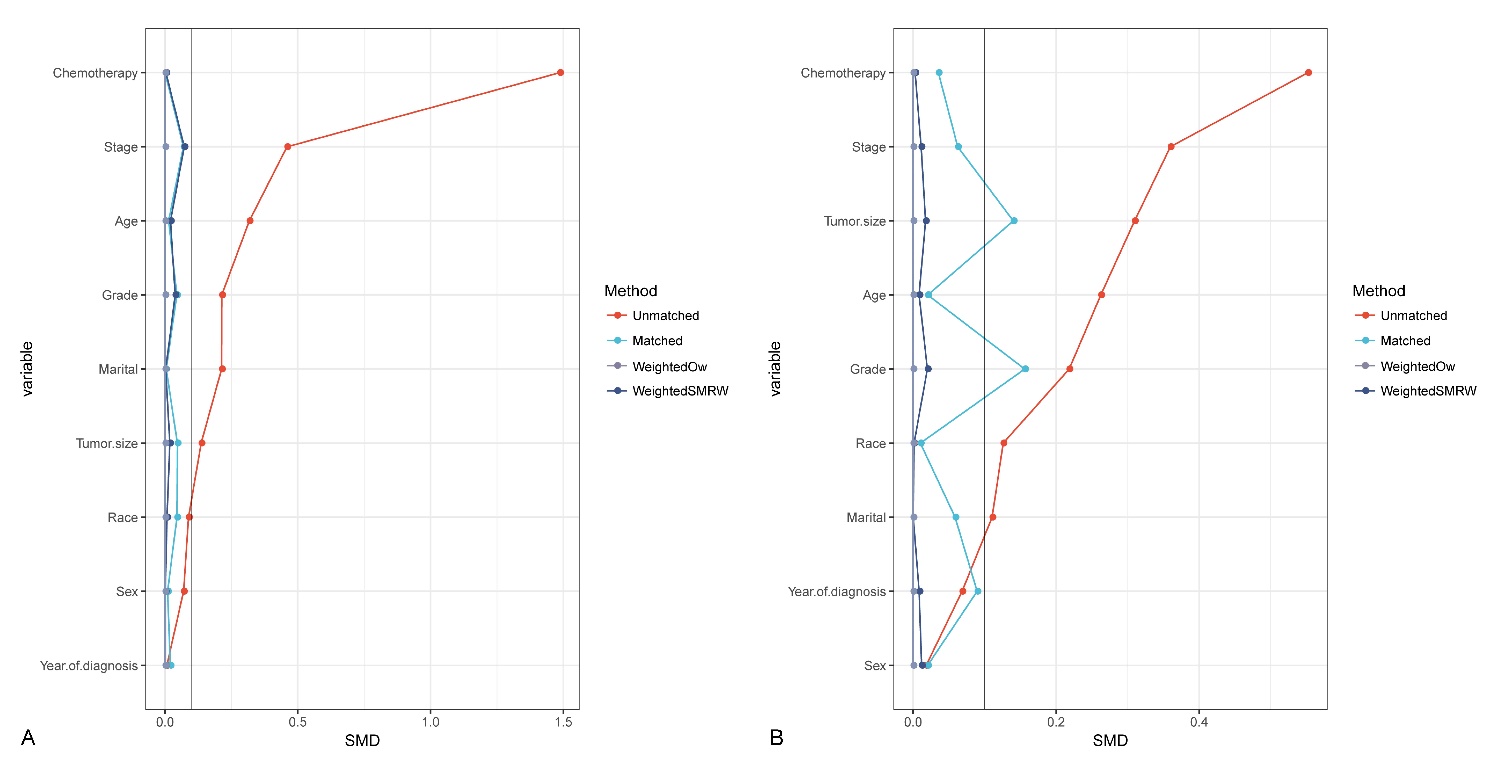


**Supplemental Figure 2.** SMD values in the model after PSM, SMRW and OW between the adjuvant treatment group (A) and palliative treatment group (B). Adjusted for potentially confounding factors in these models (i.e., age, sex, year of diagnosis, marital status, race, stage, tumor size, and chemotherapy status).
